# Shortened red blood cell age in patients with end-stage renal disease who were receiving haemodialysis: a cross-sectional study

**DOI:** 10.1186/s12882-020-02078-z

**Published:** 2020-09-29

**Authors:** Koichiro Matsumura, Toshika Okumiya, Tetsuro Sugiura, Nobuyuki Takahashi, Yoshihiro Yamamoto, Sanae Kikuchi, Kenichi Fujii, Munemitsu Otagaki, Ichiro Shiojima

**Affiliations:** 1 Department of Medicine II, Kansai Medical University, 10-15 Fumizono-cho, Moriguchi, 5708507 Japan; 2 Department of Biomedical Laboratory Sciences, Faculty of Health Sciences, Kumamoto University, Kumamoto, Japan

**Keywords:** Anaemia, Haemodialysis, Peritoneal dialysis, Red blood cell age

## Abstract

**Background:**

The causes of anaemia in patients with end-stage renal disease include a relative deficiency in erythropoietin production and complex clinical conditions. We aimed to investigate the underlying mechanisms of anaemia in patients with end-stage renal disease who were undergoing maintenance dialysis by measuring erythrocyte creatine levels.

**Methods:**

In a cross-sectional study, we evaluated 69 patients with end-stage renal disease who were receiving haemodialysis (*n* = 55) or peritoneal dialysis (*n* = 14). Erythrocyte creatine level, a quantitative marker of mean red blood cell (RBC) age, was measured.

**Results:**

The mean RBC age was significantly shorter in the haemodialysis group than in the peritoneal dialysis group (47.7 days vs. 59.8 days, *p* < 0.0001), although the haemoglobin levels were comparable between the groups. A Spearman correlation coefficient analysis revealed that shortened RBC age positively correlated with transferrin saturation (*r* = 0.54), ferritin level (*r* = 0.47), and haptoglobin level (*r* = 0.39) but inversely related with reticulocyte (*r* = − 0.36), weekly doses of erythropoiesis-stimulating agents (ESAs; *r* = − 0.62), erythropoietin resistance index (*r* = − 0.64), and intradialytic ultrafiltration rate (*r* = − 0.32).

**Conclusions:**

Shortened RBC age was observed in patients who were receiving maintenance haemodialysis and was associated with iron deficiency, greater haptoglobin consumption, higher ESA requirements, and poor erythropoietin responsiveness, as well as with greater intradialytic fluid extraction.

## Background

Anaemia is a common complication in patients with end-stage renal disease and is associated with poor long-term survival [1]. The causes of anaemia in end-stage renal disease include a relative deficiency in erythropoietin production and complex clinical conditions such as iron deficiency, inflammation, and haemolysis [2]. However, the development of anaemia related to complex clinical conditions in end-stage renal disease, especially haemodialysis, is undetermined. Labelling erythrocytes with radio-active chromium (^51^Cr) is the standard method for estimating RBC age, which requires exclusive equipment for radio-active materials and a prolonged examination period with serial blood withdrawals from the patients [3]. Compared with the ^51^Cr-labelling method, erythrocyte creatine is a simple, rapid, and economically favourable marker that can be measured using a single blood sample examination. Erythrocyte creatine level is considered a quantitative marker of mean RBC age because young RBCs contain substantially higher creatine levels than older RBCs, and creatine contents in RBCs decrease gradually with advancing cell age; an elevated erythrocyte creatine level reflects a shortened RBC age [4–6]. Moreover, in contrast to reticulocyte levels, which reflect present erythropoiesis, erythrocyte creatine levels reflect average or cumulative erythropoiesis up to the present [5, 7]. Accordingly, we used erythrocyte creatine level to elucidate the incidence and underlying mechanisms associated with the development of anaemia in patients with end-stage renal disease who were receiving maintenance haemodialysis or peritoneal dialysis.

## Methods

### Study design

We assessed haemolysis in outpatients with end-stage renal disease who were recruited from the dialysis unit of Kansai Medical University Hospital from May to November 2019. Patients aged ≥20 years who had been receiving maintenance haemodialysis 3 times a week or peritoneal dialysis therapy for at least 6 months were included in this cross-sectional study. The exclusion criteria were as follows: patients undergoing both haemodialysis and peritoneal dialysis, patients with a bleeding event within the last 3 months, blood transfusion within the last 3 months, concurrent malignancy, haemolytic disease, and mechanical heart valves. The study protocol was approved by the ethics committee of Kansai Medical University (No. 2018233) and registered in the University Hospital Medical Information Network (UMIN) clinical trial registry (URL: https://www.umin.ac.jp/ctr/, Unique Identifier: UMIN000036418). All the patients provided written informed consent, and the investigation conformed to the principles outlined in the Declaration of Helsinki.

### Haemodialysis and peritoneal dialysis

Haemodialysis was performed via native arteriovenous fistulas with a dual plastic needle and 16-gauge cannula. The patients in the haemodialysis group uniformly received a dialysate (D-dry, Nikkiso Co., Ltd., Tokyo, Japan) and an anticoagulant with heparin sodium. Bolus heparin sodium 500 to 1000 units was intravenously administrated at the start of haemodialysis, followed by continuous administration of 500 to 1000 units maintain the pre-haemodialysis activated partial thromboplastin time at 1.5 to 2 times higher than its upper level. The dialysate temperature of extracorporeal circulation was strictly maintained at 36 °C–38 °C. Nocturnal intermittent peritoneal dialysis (Baxter Healthcare, Tokyo, Japan) was performed in all the patients in the peritoneal dialysis group. Evaluation and treatment of anaemia, including erythropoiesis-stimulating agents (ESAs) and iron therapy, were prescribed in accordance with the Kidney Disease: Improving Global Outcomes Clinical Practise Guideline 2012 [8]. Iron administration therapy with an intravascular supplement (40 mg of iron/week) was administrated to the haemodialysis group; and oral supplement (100 mg of iron/day) to the peritoneal dialysis group. ESA therapies with darbepoetin alfa and epoetin beta pegol were administrated to the haemodialysis and peritoneal dialysis groups, respectively. Blood flow (mL/min) and intradialytic ultrafiltration rates (mL/h/kg) were measured to assess the haemodialysis condition, which was calculated as the average of the values from 3 consecutive haemodialysis sessions. One of the following dialysis membranes was used in the haemodialysis group by dialysis unit physicians: cellulose (FB-Uβ, Nipro Corporation, Osaka, Japan), polysulfone, (ABH-PA, Asahi Kasei Corporation, Tokyo, Japan; APS-EA, Asahi Kasei Corporation; NV-X, Toray Medical Co., Ltd., Tokyo, Japan; NVF-H, Toray Medical Co., Ltd.; and VPS-VA, Asahi Kasei Corporation), polyethersulfone (MFX-S, Nipro Corporation; PES-D, Nipro Corporation), polymethylmethacrylate (NF-H, Toray Medical Co., Ltd.), or acrylonitrile-co-methallyl sulphonate (H12–4000, Baxter, Tokyo, Japan).

### Measurements

Body weight was measured before and after dialysis in the haemodialysis group. In the peritoneal dialysis group, body weight was measured after discarding the dialysate from the peritoneal cavity. After enrolment, blood samples were drawn from all the patients to examine the level of erythrocyte creatine, haemolytic markers (reticulocyte, haptoglobin, and lactate dehydrogenase), and other laboratory parameters (haemoglobin, haematocrit, albumin, transferrin saturation, and ferritin). Blood samples were obtained immediately before the patients received haemodialysis. A weekly ESA dose was administered as a darbepoetin alfa equivalent dose. ESA was converted using the following formula: darbepoetin alfa (μg) = epoetin beta pegol (μg) × 0.8 = epoetin (U) × 200, in accordance with previous reports [9, 10]. ESA responsiveness was assessed using an erythropoietin resistance index, which was calculated using the following formula: erythropoietin resistance index (U/kg/week/g/dL) = weekly dose of epoetin (U/week)/(body weight [kg] × haemoglobin level [g/dL]) [11]. Post-haemodialysis weight was measured as a body weight in the haemodialysis group.

### Erythrocyte creatine

Creatine is packed in erythrocytes and irreversibly decreases in amount over time. By measuring the erythrocyte creatine level, the mean RBC age can be calculated [4]. Erythrocyte creatine was measured enzymatically in accordance with previous reports [12]. Briefly, blood samples were collected in ethylenediaminetetraacetic acid-containing tubes and centrifuged to remove the plasma and buffy coat. After lysis and deproteinisation of packed erythrocytes, the supernatant was obtained by centrifugation and filtration. The creatine concentration in the supernatant was measured using the enzymatic assay method. The mean RBC age (days) was − 22.84 × log_e_ (erythrocyte creatine) + 65.83 [6]. Erythrocyte creatine levels represent the average or cumulative erythropoiesis up to the present. Therefore, erythrocyte creatine levels are indicative of chronic rather than an acute haemolytic conditions. The RBC ages in 305 healthy subjects in our previous study were extracted as healthy controls [5, 6].

### Statistical analyses

Continuous variables are presented as medians and interquartile ranges and categorical variables as numbers and percentages. Differences between the 2 groups were analysed using the Wilcoxon rank-sum test for continuous variables and the chi-squared test for categorical variables. The relationship between the clinical covariates and erythrocyte creatine level was examined using a Spearman correlation analysis. A *p* value of < 0.05 was considered significant. The JMP 14.2.0 software (SAS Institute Inc., Cary, NC, USA) was used for all the statistical analyses.

## Results

A total of 80 outpatients aged > 20 years were included in the study. Of these patients, those who were undergoing both haemodialysis and peritoneal dialysis (*n* = 6) and had a bleeding event within the last 3 months (*n* = 1), mechanical heart valves (*n* = 2), and who did not provide written informed consent (*n* = 2) were excluded. Finally, 55 patients who were receiving haemodialysis and 14 who were receiving peritoneal dialysis were included in the final analysis.

No significant differences in patient characteristics were found between the groups (Table 1). Although no significant difference in haemoglobin level was observed between the groups, the haemodialysis group had significantly lower transferrin saturation and ferritin levels than the peritoneal dialysis group. The weekly ESA dose and erythropoietin resistance index were significantly higher in the haemodialysis group than in the peritoneal dialysis group.

**Table 1:** Patients’ profiles

	Haemodialysis (*n* = 55)	Peritoneal dialysis (*n* = 14)	*p* Value
**Age (years)**	71 (63–79)	70 (60–79)	0.83
**Male sex**	33 (60)	7 (50)	0.45
**Body mass index (kg/m**^**2**^**)**	21.3 (19.8–24.3)	23.4 (21.3–24.5)	0.12
**Hypertension**	47 (85)	13 (93)	0.46
**Diabetes mellitus**	32 (58)	5 (36)	0.13
**Prior cardiovascular disease**	14 (25)	3 (21)	0.76
**Laboratory data**			
**Haemoglobin (g/dL)**	11.0 (10.5–11.7)	11.0 (10.5–11.8)	0.78
**Haematocrit (%)**	33.9 (32.1–36.5)	33.8 (33.1–35.9)	0.70
**Albumin (g/dL)**	3.5 (3.3–3.8)	3.5 (3.1–3.7)	0.35
**High-sensitive CRP (mg/dL)**	0.06 (0.04–0.29)	0.09 (0.02–0.43)	0.82
**iPTH (pg/mL)**	139 (103–190)	203 (150–240)	0.04
**Transferrin saturation (%)**	22 (16–28)	37 (24–43)	0.008
**Ferritin (ng/mL)**	50 (29–71)	119 (105–161)	< 0.0001
**Reticulocyte (%)**	1.7 (1.4–2.1)	1.3 (1.0–1.7)	0.06
**Haptoglobin (mg/dL)**	78 (38–121)	96 (76–184)	0.04
**Lactate dehydrogenase (U/L)**	194 (176–212)	184 (156–225)	0.50
**Iron supplement**	18 (33)	3 (21)	0.40
**Erythropoiesis-stimulating agent (μg/week)**	30 (10–45)	12 (10–20)	0.02
**Erythropoietin resistance index**	9.4 (3.4–14.1)	3.9 (2.7–7.5)	0.02

Data are presented as median (25th to 75th percentiles), or number (%)

*CRP* C-reactive protein, *iPTH* intact parathyroid hormone

**Fig. 1:**
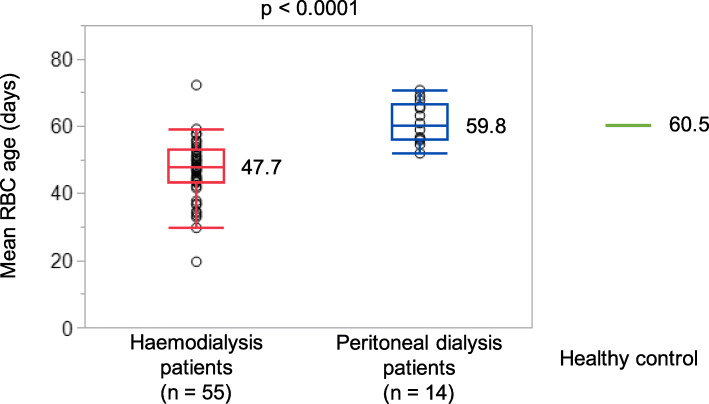
Comparison of mean RBC age. The mean RBC age in the healthy controls is shown as a green line. The box for the 2 groups represents the interquartile range (25th–75th percentile), and the horizontal line in each box represents the median value. RBC: red blood cell

The RBC ages are shown in Fig. 1. The haemodialysis group had significantly shorter RBC age than the peritoneal dialysis group (47.7 [43.2–52.8] days vs. 59.8 [56.0–66.2] days, *p* < 0.0001). The RBC age in the healthy controls was 60.5 days, which was comparable with that in the peritoneal dialysis group.

When the patients in the haemodialysis group were stratified by median RBC age (47.7 days), those with shortened RBC age had lower transferrin saturation, ferritin, and haptoglobin levels than those with preserved RBC age, despite the higher iron administration rate (Table 2). The weekly ESA dose, ESA resistance index, and intradialytic ultrafiltration rate were all significantly higher in the patients with shortened RBC age than in those with preserved RBC age. On the other hand, no significant difference in the type of dialysis membranes was found between the groups.

**Table 2:** Comparison of clinical characteristics of the patients who were receiving haemodialysis according to median RBC age

	Preserved RBC age (*n* = 27)	Shortened RBC age (*n* = 28)	*p* Value
**Age (years)**	72 (67–79)	71 (61–78)	0.32
**Male sex**	16 (59)	17 (61)	0.91
**Body mass index (kg/m**^**2**^**)**	21.3 (19.8–23.6)	21.3 (19.5–25.2)	0.89
**Laboratory data**			
**Haemoglobin (g/dL)**	10.9 (10.6–11.4)	11.1 (10.3–11.9)	0.91
**Haematocrit (%)**	33 (31–35)	35 (33–37)	0.09
**Albumin (g/dL)**	3.6 (3.3–3.8)	3.5 (3.3–3.8)	0.97
**High-sensitive CRP (mg/dL)**	0.06 (0.04–0.43)	0.06 (0.04–0.26)	0.67
**iPTH (pg/mL)**	135 (98–182)	143 (104–197)	0.55
**Transferrin saturation (%)**	26 (19–39)	18 (14–26)	< 0.01
**Ferritin (ng/mL)**	57 (36–122)	43 (20–65)	0.04
**Reticulocyte (%)**	1.7 (1.2–2.1)	1.7 (1.4–2.1)	0.72
**Haptoglobin (mg/dL)**	91 (61–139)	49 (25–114)	< 0.01
**Lactate dehydrogenase (U/L)**	194 (176–207)	198 (177–214)	0.72
**Iron supplement**	5 (19)	13 (46)	0.03
**Erythropoiesis-stimulating agent (μg/week)**	20 (10–30)	33 (20–60)	0.004
**Erythropoietin resistance index**	5.0 (2.8–11.8)	11.4 (6.7–20.8)	0.01
**Quantity of blood flow (mL/min)**	220 (200–250)	200 (200–245)	0.53
**Intradialytic ultrafiltration rate (mL/h/kg)**	7.3 (6.0–9.3)	9.3 (7.0–12.7)	0.02
**Dialysis membranes**			
**Cellulose**	11 (41)	9 (32)	0.51
**Polysulfone**	6 (22)	10 (36)	0.27
**Polyethersulfone**	8 (30)	7 (25)	0.70
**Polymethylmethacrylate**	1 (4)	0 (0)	0.23
**Acrylonitrile-co-methallyl sulfonate**	1 (4)	2 (7)	0.57
**Heparin dosage (units/session)**	2850 (2750–3825)	3205 (2800–3825)	0.52

Data are presented as median (25th to 75th percentiles), or number (%)

*CRP* C-reactive protein, *iPTH* intact parathyroid hormone, *RBC* red blood cell

To investigate the correlation of the clinical covariates with mean RBC age, a Spearman correlation coefficient analysis was performed (Table 3). Transferrin saturation, ferritin, and haptoglobin level positively correlated with RBC age, whereas reticulocyte, weekly ESA dose, erythropoietin resistance index, and intradialytic ultrafiltration rate negatively correlated with RBC age.

**Table 3:** Spearman correlations of the clinical covariates for mean RBC age

	*r*	*p* Value
**Age (years)**	0.03	0.78
**Haemoglobin (g/dL)**	0.02	0.85
**High-sensitive CRP (mg/dL)**	−0.02	0.88
**iPTH (pg/mL)**	0.08	0.52
**Transferrin saturation (%)**	0.54	< 0.0001
**Ferritin (ng/mL)**	0.47	< 0.0001
**Reticulocyte (%)**	−0.36	0.002
**Haptoglobin (mg/dL)**	0.39	0.001
**Erythropoiesis-stimulating agent (μg/week)**	−0.62	< 0.0001
**Erythropoietin resistance index**	−0.64	< 0.0001
**Quantity of blood flow (mL/min)**	0.07	0.59
**Intradialytic ultrafiltration rate (mL/h/kg)**	−0.32	0.02

*RBC* red blood cell

## Discussion

In the present study, RBC age was measured in patients with end-stage renal disease who were receiving dialysis therapy. We found a significant shortening of RBC age in the patients who were receiving haemodialysis as compared with those who were receiving peritoneal dialysis, despite that no significant differences in haemoglobin level was found between the 2 groups. Moreover, the Spearman correlation coefficient revealed that shortened RBC age was associated with iron deficiency, greater haptoglobin consumption, higher ESA requirements, and poor ESA responsiveness, as well as with higher intradialytic ultrafiltration rate.

A prospective small study that used radio-active chromium (^51^Cr), investigated the shortening of RBC age in patients with end-stage renal disease who were receiving haemodialysis and peritoneal dialysis, and in healthy volunteers with preserved glomerular filtration rate (> 60 mL/min/1.73m^2^) [13]. RBC age was shortened by 20% in the patients with end-stage renal disease as compared with the healthy volunteers, but no significant difference in RBC age was found between the haemodialysis and peritoneal dialysis groups. Owing to the small number of patients in each group and the lack of haemolytic markers, the authors did not analyse the mechanisms of the RBC age shortening in the patients who were receiving dialysis. In our study, shortening of RBC age was observed in the haemodialysis group as compared with the peritoneal dialysis group, whereas RBC age was comparable between the peritoneal dialysis and healthy control groups. Although this discrepancy was not fully elucidated, our study shows that the patients who were receiving haemodialysis had greater iron consumption and required higher ESA dosages than those who were receiving peritoneal dialysis, indicating that the patients who were receiving haemodialysis were accompanied by absolute or functional iron deficiency. RBC age shortening leads to systemic tissue hypoxia, which stimulates endogenous erythropoietin production and enhances iron availability [1]. Persistent RBC age shortening is attributable to absolute or functional iron deficiency and relative ESA hyporesponsiveness. Therefore, iron deficiency and higher ESA dosage requirement in patients receiving haemodialysis indicate persistent RBC age shortening.

The haptoglobin level was significantly lower in the patients who were receiving haemodialysis than in those who were receiving peritoneal dialysis. Meyer C et al. investigated haemodialysis-induced haemolysis using free haemoglobin levels [14]. They calculated free haemoglobin level before and after haemodialysis and found a significant increase after haemodialysis. These data suggest that haemodialysis-induced haemolysis is one of the underlying mechanisms of shortened RBC age because the mechanical stress caused by the flow resistance and turbulence during the extracorporeal circuit often contributes to haemodialysis-induced haemolysis. Toshner et al. investigated alterations in haptoglobin and lactate dehydrogenase levels between before and 8 h after haemodialysis in 12 patients, and found that both parameters did not change during the period [15]. They concluded that RBC damage due to mechanical stress of the extracorporeal circuit was a negligible contributing factor to persistent anaemia. However, the baseline haptoglobin level was quite heterogeneous (9 to 210 mg/dL) among the study population. This difference in haptoglobin level was also observed in our study. Moreover, the haptoglobin level was significantly lower in the patients with shortened RBC age in the haemodialysis group than in those with preserved RBC age, indicating a potential relationship between haptoglobin level and shortened RBC age.

A 16-gauge plastic needle was used in this study, and the median blood flow ranged from 200 to 220 ml/min. A previous study reported no significant differences in the level of haemolysis markers between using a 15-gauge needle at a blood flow rate of 400 ml/min and a 16-gauge needle at a blood flow rate of 300 ml/min [16]. Likewise, no significant differences in the levels of haemolytic markers were observed between using a 14-gauge needle group at a 500-ml/min blood flow rate and using a 17-gauge needle at a 250-ml/min blood flow rate, which suggests that the size of the puncture needle does not affect haemolysis [17]. Greater intradialytic ultrafiltration rate, not increased blood flow rate, was observed in the patients with shortened RBC age in the haemodialysis group, which suggests that greater ultrafiltration volume through the dialysis membrane rather than intraluminal blood velocity was one of the underlying mechanisms associated with the increased shear stress of the circulating erythrocytes that causes haemolysis.

The patients with shortened RBC age in the haemodialysis group had greater iron deficiency despite the higher rate of iron supplementation than those with preserved RBC age. Although adequate intravenous iron therapy has been widely accepted to optimise ESA responsibility, a recent European national study demonstrated that intravenous or oral iron supplementation was used only in 19% of patients with end-stage renal disease [18]. Adequate iron therapy may improve ESA responsiveness, especially in patients with shortened RBC age who are receiving haemodialysis.

In addition to the high prevalence of known risk factors of cardiovascular disease, haemolysis-associated endothelial dysfunction has been reported [14, 19, 20]. The release of free haemoglobin induced by haemolysis scavenges and reduces the bioavailability of nitric oxide, which leads to impaired vascular endothelial function. Impaired endothelial function due to haemodialysis-induced haemolysis could lead to an increased risk of cardiovascular complications. Several complex clinical conditions, including iron deficiency, ESA responsiveness, and haemolysis, are involved in the development of anaemia in patients with end-stage renal disease. Therefore, correct recognition of shortened RBC age is important to reduce the risk of future unfavourable cardiovascular events in patients receiving maintenance haemodialysis.

### Limitations

Three limitations of the present study should be noted. First, this study included a relatively small sample size, and the number of patients in the haemodialysis and peritoneal dialysis group were discrepant. Therefore, investigations using a larger sample size are needed to verify our results. Second, tissue hypoxia due to low cardiac output, hypotension, or anaemia leads to increased endogenous erythropoietin production and enhanced iron availability. Increased endogenous erythropoietin production contributes to accelerated erythropoiesis, which results in higher erythrocyte creatine values. Third, we excluded patients with unstable systemic circulation, such as those who had bleeding events, received blood transfusion, had a malignant disease, or were introduced to dialysis within 6 months, but erythrocyte creatine level has potential limitations defining RBC age in patients with end-stage renal disease.

## Conclusions

Shortened RBC age was observed in patients who were receiving maintenance haemodialysis. Iron deficiency, erythropoietin hyporesponsiveness, haemolysis, and greater intradialytic fluid extraction were related to shortened RBC age.

## Data Availability

The datasets generated during and/or analysed during the present study are available from the corresponding author upon reasonable request.

## Abbreviation

RBC: Red blood cell
